# Bax Inhibitor-1 regulates hepatic lipid accumulation via ApoB secretion

**DOI:** 10.1038/srep27799

**Published:** 2016-06-14

**Authors:** Hwa Young Lee, Geum-Hwa Lee, Kashi Raj Bhattarai, Byung-Hyun Park, Seung-Hoi Koo, Hyung-Ryong Kim, Han Jung Chae

**Affiliations:** 1 Department of Pharmacology, School of Medicine, Chonbuk National University, Jeonju 560-182, Korea; 2 Department of Biochemistry, School of Medicine, Chonbuk National University, Jeonju 560-182, Korea; 3 Division of Life Sciences, Korea University, 145 Anam-Ro, Seongbuk-Gu, Seoul, 136-713, Korea; 4 Department of Dental Pharmacology, School of Dentistry, Wonkwang University, Iksan, 570-749, Korea

## Abstract

In this study, we explored the effects of Bax Inhibitor-1 (BI-1) on ApoB aggregation in high-fat diet (HFD)-induced hepatic lipid accumulation. After 1 week on a HFD, triglycerides and cholesterol accumulated more in the liver and were not effectively secreted into the plasma, whereas after 8 weeks, lipids were highly accumulated in both the liver and plasma, with a greater effect in BI-1 KO mice compared with BI-1 WT mice. ApoB, a lipid transfer protein, was accumulated to a greater extent in the livers of HFD-BI-1 KO mice compared with HFD-BI-1 WT mice. Excessive post-translational oxidation of protein disulfide isomerase (PDI), intra-ER ROS accumulation and folding capacitance alteration were also observed in HFD-BI-1 KO mice. Higher levels of endoplasmic reticulum (ER) stress were consistently observed in KO mice compared with the WT mice. Adenovirus-mediated hepatic expression of BI-1 in the BI-1 KO mice rescued the above phenotypes. Our results suggest that BI-1-mediated enhancement of ApoB secretion regulates hepatic lipid accumulation, likely through regulation of ER stress and ROS accumulation.

Nonalcoholic fatty liver disease (NAFLD) is characterized by excessive lipid accumulation in the liver in the absence of ‘significant’ alcohol consumption, and can progress to nonalcoholic steatohepatitis, fibrosis, cirrhosis, and hepatocellular carcinoma^1^. NAFLD is clearly associated with features of metabolic syndrome, including obesity, type II diabetes, hypertension, and dyslipidemia. Hepatic steatosis is considered to be the first stage of NAFLD and often leads to more severe complications, including steatohepatitis, cirrhosis, and hepatocellular carcinoma^2^,^3^,^4^. A growing number of studies examining the mechanism of hepatic steatosis have focused on the causative role of ER stress.

Events that disturb ER protein folding and induce the unfolded protein response (UPR) include alterations in the redox state, calcium equilibrium, and protein degradation. Similarly, the accumulation of fatty acids or triglycerides is related to alterations of secretory apolipoproteins such as ApoB, which can also induce the UPR and cause hepatic steatosis. The secretion of ApoB-containing lipoproteins involves both co- and post-translational processes. Unassembled or aberrantly expressed ApoB retained in the ER is typically degraded, and when under mild physiological stress, the degradation process is highly activated as an adaptive response involving both ER-resident molecular chaperones such as calnexin and calreticulin, and ER proteases such as ER60^5^,^6^. However, when pathological ER stress conditions are not regulated by the adaptive response, the physiological degradation machinery does not function efficiently, leading to the accumulation of unfolded proteins, including ApoB^7^. During this type of ER stress, hepatic lipid synthesis and secretion may also be affected by alterations in the folding process of secretory ApoB proteins^8^. Therefore, it is necessary to study ER stresses to determine how to control pathological phenomena such as hepatic steatosis.

The anti-apoptotic protein Bax inhibitor-1 (BI-1)^9^ was originally identified as an inhibitor of ER stress-induced apoptosis. BI-1 contains six transmembrane regions and localizes to ER membranes. Its cytoprotective function is well-conserved in plants and mammals^10^,^11^. Based upon these observations, we examined the potential regulatory effects of BI-1 on hepatic dyslipidemia. We found that BI-1 significantly inhibited hepatic lipid accumulation *in vivo*. Our results suggest a regulatory mechanism of BI-1 in hepatic dyslipidemia.

## Results

### BI-1 regulates hepatic lipid metabolism on short and long-term high-fat diets

**Figure 1:**
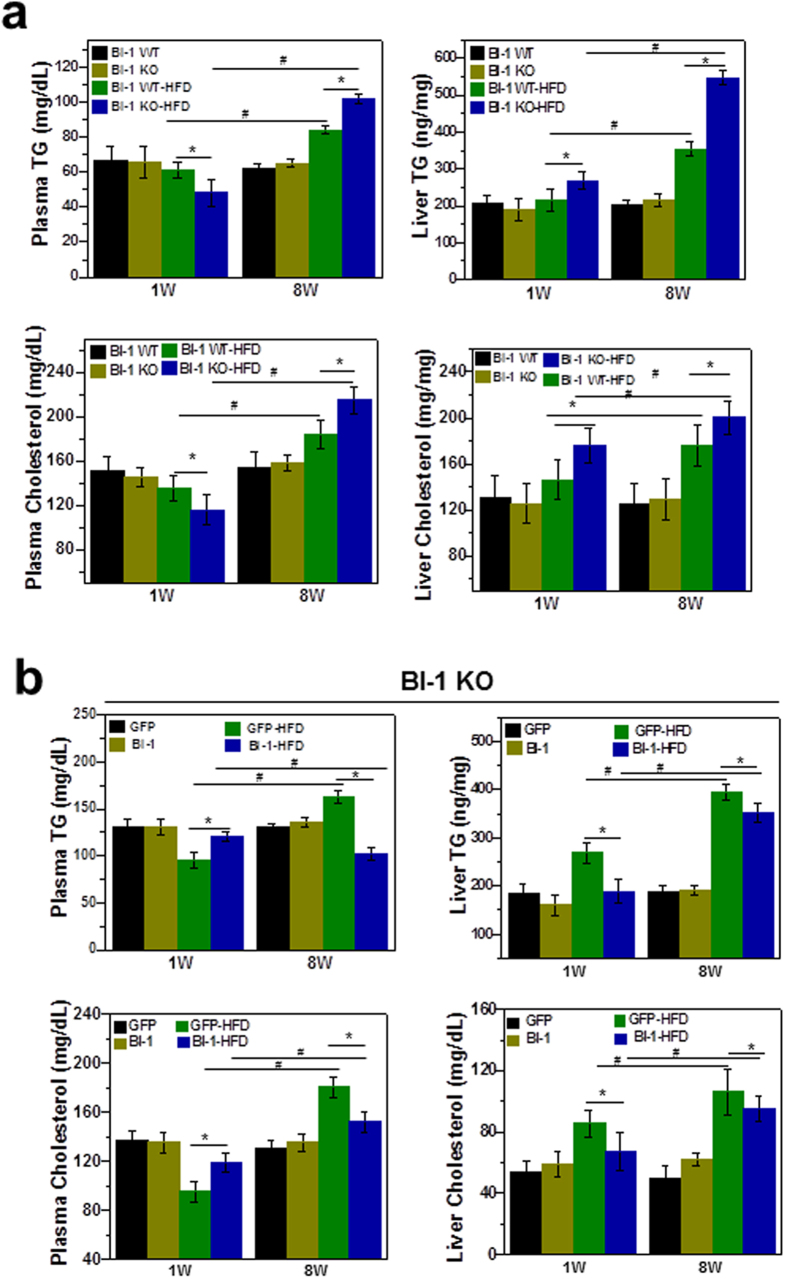
BI-1 regulates hepatic lipid accumulation in mice fed a high-fat diet. BI-1 WT and BI-1 KO mice (8 weeks old, n = 5) were fed with HFD for 1- and 8-weeks. (**a**) Six hour-fasting plasma and liver triglyceride (upper) and cholesterol levels (lower) were measured at 1- and 8-week HFD-fed BI-1 WT and BI-1 KO mice, as described in Experimental Procedures. (**b**) Six-hour fasting plasma and liver triglyceride (upper) and cholesterol levels (lower) for 1- and 8-weeks HFD-fed BI-1 KO mice that were infected with GFP control virus and BI-1-expressing virus, separately (Mean ± SEM; n = 5, *p < 0.05, ^#^*p* < 0.001). TG; triglyceride, HFD; high-fat diet, WT; wild-type, KO; knock-out, GFP; green fluorescent protein.

**Figure 2:**
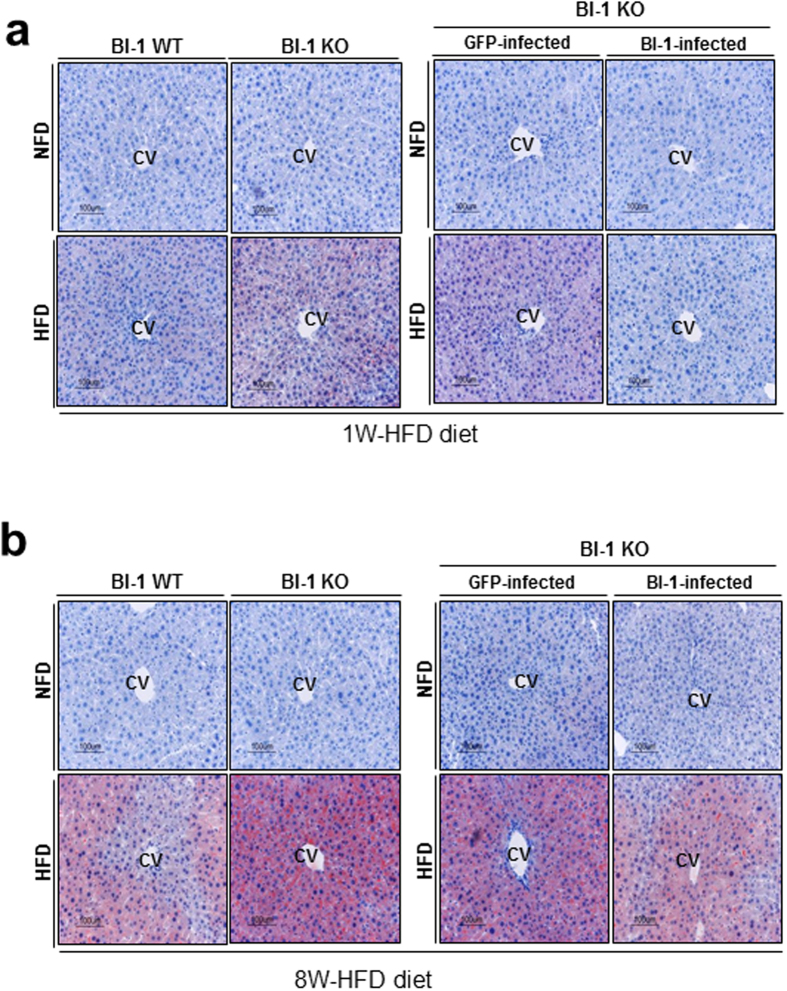
BI-1 regulates hepatic lipid accumulation, showing lipid staining. (**a**) BI-1 WT and BI-1 KO mice (8 weeks old, n = 5) were fed with HFD for 1-week and separately, 1-week-HFD-fed BI-1 KO mice were infected with GFP control virus and BI-1-expressing virus (right). (**b**) BI-1 WT and BI-1 KO mice (8 weeks old, n = 5) were fed with HFD for 8-weeks and separately, 8-weeks-HFD-fed BI-1 KO mice were infected with GFP control virus and BI-1-expressing virus (right). Oil Red O staining was performed. Images of liver samples were obtained at 200× original magnification. CV; central vein, NFD; normal-fat diet, HFD; high-fat diet, WT; wild-type, KO; knock-out, GFP; green fluorescent protein.

To examine the effects of HFD on liver injury, BI-1 wild type (WT) and knock-out (KO) mice were fed a high-fat diet (HFD) for 1 and 8 weeks. Serum alanine transaminase (ALT) and aspartate aminotransferase (AST) levels were markedly elevated both in BI-1 WT and KO mice after 8 weeks on a HFD, and ALT and AST levels were more significantly increased in KO than WT mice (Supplementary Fig. 1a). Increased levels of serum ALT and AST in HFD-BI-1 KO mice were significantly reduced by infection with adenovirus BI-1 in contrast to infection with the adenovirus GFP control (Supplementary Fig. 1b). To investigate the ability of BI-1 to regulate lipid accumulation, BI-1 WT and KO mice were fed high-fat diet (HFD) to induce hepatic lipid accumulation. The levels of triglycerides (TG) and cholesterol were measured in plasma and liver lysates of HFD-BI-1 WT or KO mice at 1- and 8-weeks. In BI-1 KO mice, plasma TG level was significantly decreased compared with BI-1 WT mice in the 1-week HFD group (Fig. 1a). However, the plasma TG level was highly increased both in BI-1 WT and KO mice after 8 weeks on a HFD, and TG levels were relatively higher in the KO group compared with WT mice. Liver TG was significantly increased in the 1-week-HFD BI-1 KO mice, whereas liver TG was more highly accumulated in the 8 week-feeding BI-1 KO mice than the counter-mice. Plasma and liver cholesterol level showed similar pattern to the TG level. The changed levels of plasma and liver TG in HFD-BI-1 KO mice were recovered by infection with adenoviral BI-1, compared with infection with adenoviral GFP control (Fig. 1b). The levels of plasma and liver cholesterol were also recovered by viral expression of BI-1 in HFD-fed BI-1 KO mice. Consistently, a more significant increase in the accumulation of lipid droplets was observed in 1- and 8-week HFD-fed BI-1 KO liver, compared with the 1- and 8-week HFD-fed BI-1 WT liver, as determined by oil red O staining (Fig. 2a,b). In BI-1 KO mice, the accumulation of lipid droplets in hepatocytes was clearly reduced in BI-1 virus-infected mice under both 1- and 8-week HFD conditions.

**Figure 3:**
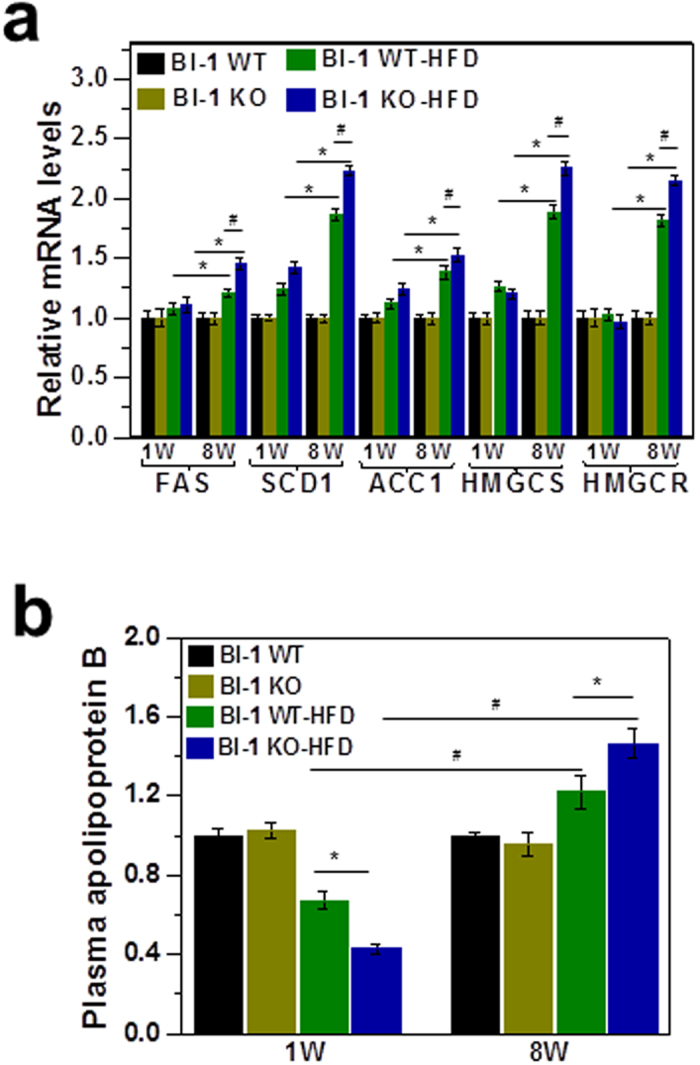
BI-1 regulates fatty acid synthesis gene expression and ApoB lipoprotein secretion. (**a**) BI-1 WT and BI-1 KO mice (8 weeks old, n = 5) were fed with HFD for 1- and 8-weeks. After mRNA isolation, the transcription of genes involved in triglyceride and cholesterol biosynthesis was measured in the livers of HFD-fed mice. The mRNA levels of genes encoding FAS, SCD1, ACC1, HMGCS, and HMGCR in the livers of the mice were determined by real-time RT-PCR. (**b**) Immunoblot analysis of plasma ApoB from 1- and 8-weeks HFD fed BI-1 WT or KO mice. The relative levels of plasma ApoB were measured compared to the mean levels in mice fed a normal diet. (Mean ± SEM; n = 5, *p < 0.05, ^#^*p* < 0.001). HFD; high-fat diet, FAS; fatty acid synthase, SCD1; stearoyl CoA desaturase 1, ACC1; acetyl-CoA carboxylase, HMGCS; 3′-hydroxylmethyl glutaryl coenzyme A synthetase, HMGCR; 3′-hydroxylmethyl glutaryl coenzyme A reductase.

To assess differences in hepatic lipid accumulation and plasma lipid after 1- and 8-weeks of HFD feeding, the expression of enzymes involved in fatty acid and TG synthesis was analyzed. Levels of fatty acid synthase (FAS), stearoyl CoA desaturase 1 (SCD1) and acetyl-CoA carboxylase 1 (ACC1) were highly increased in the 8-week HFD group and these levels were more significantly increased in the HFD-BI-1 KO mice compared to WT mice (Fig. 3a). However, the gene levels were slightly affected in 1-week HFD-BI-1 WT and KO mice. In addition, expression of the cholesterol biosynthesis genes, 3′-hydroxylmethyl glutaryl coenzyme A synthetase (HMGCS) and 3′-hydroxylmethyl glutaryl coenzyme A reductase (HMGCR) showed a similar pattern to fatty acid and TG synthesis gene expression profiles, indicating that lipid synthesis profiles are not a main contributor to lipid accumulation, at least not in the 1-week-BI-1 KO model.

Next, we examined apolipoprotein pathways because dysregulation of apolipoprotein secretion is related to hepatic accumulation of lipids^12^. In immunoblot analysis of plasma apolipoproteins, plasma ApoB levels were significantly decreased in BI-1 KO mice, compared with BI-1 WT mice after 1-week of HFD. There was no significant difference in ApoA-I, ApoCIII, ApoD, and ApoE levels among these groups (data not shown). However, plasma ApoB levels were rather elevated both in the 8-week HFD-BI-1 WT and KO mice (Fig. 3b), suggesting that there is a difference in apo-lipoprotein secretion profiles between 1- and 8-week HFD conditions.

### BI-1 regulates hepatic ApoB accumulation through the effective formation of disulfide bonds

**Figure 4:**
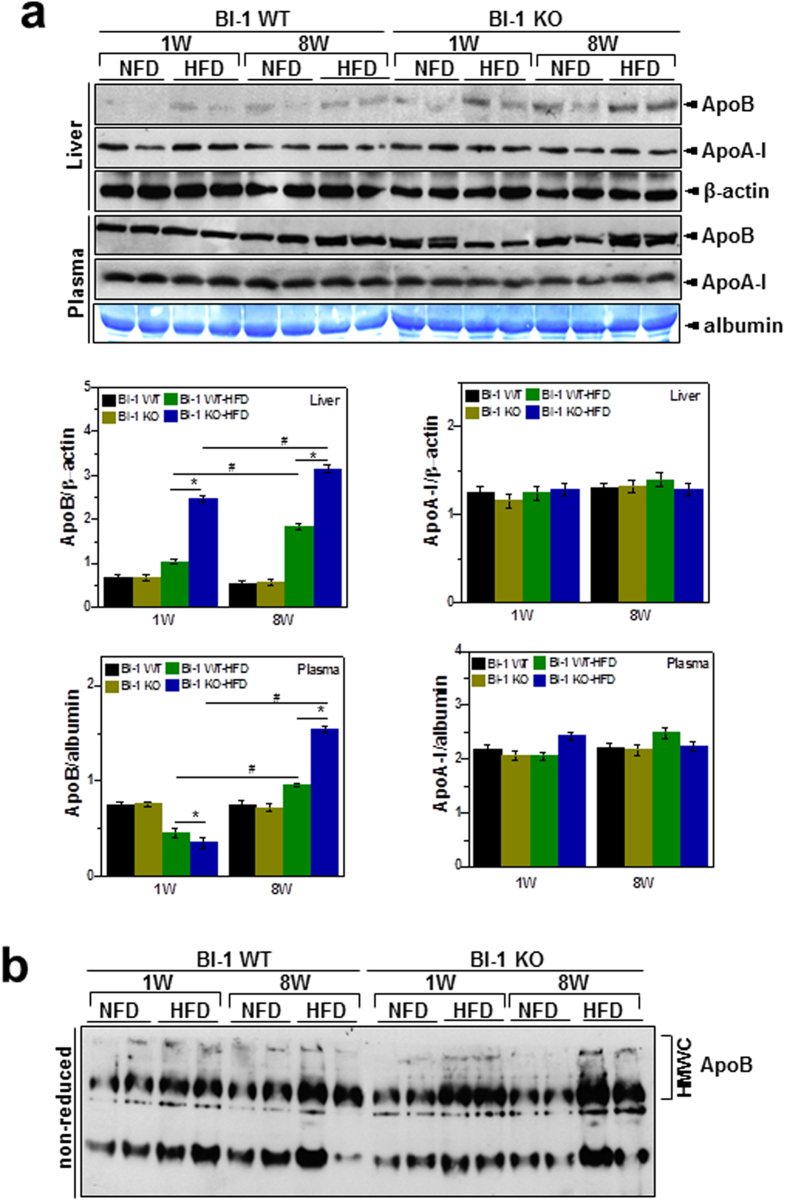
BI-1 regulates hepatic ApoB secretion. BI-1 WT and BI-1 KO mice (8 weeks old, n = 5) were fed with HFD for 1- and 8-weeks. (**a**) Liver and plasma samples were subjected to immunoblot analysis with anti-ApoB or anti-ApoA-I antibody. CBB staining (albumin) was performed as a loading control for plasma. The quantification analysis about ApoB and ApoA-I expressions was performed based upon the indicated loading control (β-actin for liver samples and albumin for plasma samples, lower) (Mean ± SEM; n = 5, *p < 0.05, ^#^*p* < 0.001). (**b**) With liver lysates from the 1- and 8-weeks-fed mice, non-reducing PAGE was applied for the confirmation of the presence of ApoB in high molecular weight complexes (HMWCs) as described in Experimental Procedures. NFD; normal-fat diet, HFD; high-fat diet, WT; wild-type, KO; knock-out, GFP; green fluorescent protein.

**Figure 5:**
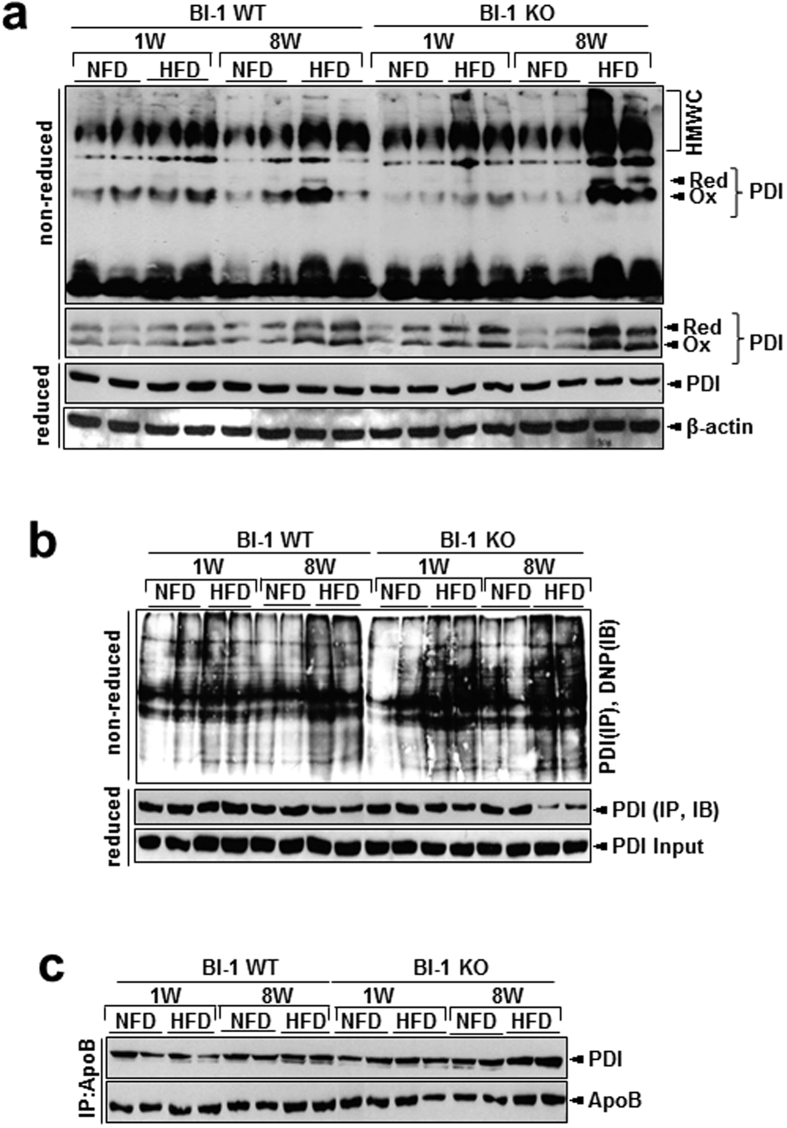
BI-1 regulates the formation of disulfide bonds during oxidative protein folding. (**a**) BI-1 WT and BI-1 KO mice (8 weeks old, n = 5) were fed with HFD for 1- and 8-weeks. Liver lysates were analyzed for the presence of PDI in high molecular weight complexes (HMWCs), using non-reducing (top and 2^nd^ panels) and reducing (3^rd^ and 4^th^ panels) gels as described in Experimental Procedures. (**b**) From the 1- and 8-weeks-HFD-fed BI-1 WT and BI-1 KO mice, livers were isolated. The liver lysates were derivatized with DNPH, and PDI was immunoprecipitated from the derivatized lysates. Immunoprecipitated PDI was run on a non-reducing gel and analyzed by western blotting using anti-DNP antibody from an OxyBlot kit. PDI, which persisted in HMWCs (unresolved, long-lasting complexes between PDI and its client proteins), was found to be excessively carbonylated. Note that the expression of the main 57-kDa band, PDI, is weaker in the immunoprecitation from HFD-fed BI-1 KO mice than that from HFD-fed BI-1 WT mice. (**c**) Liver lysates were immunoprecipitated with anti-PDI or anti-ApoB antibody and immunoblotted with anti-ApoB or anti-PDI antibody, respectively. NFD; normal-fat diet, HFD; high-fat diet, WT; wild-type, KO; knock-out, PDI; protein disulfide isomerase, DNPH; 2, 4-dinitrophenylhydrazine, DNP; dinitrophenol.

To understand the mechanism of HFD-induced alterations in hepatic lipid secretion, the expression of representative apolipoproteins, ApoA-I and ApoB, was analyzed. Expression of ApoB, but not ApoA-I, was higher in the lysates of livers from HFD-BI-1 KO mice, compared with BI-1 WT mice (Fig. 4a). In plasma, however, the level of secreted ApoB decreased especially after 1-week of HFD feeding in BI-1 KO mice. In contrast to 1-week, the level of secreted ApoB rather increased after 8-weeks in HFD-BI-1 KO mice. In ApoB protein status assay through non-native gel system, ApoB aggregates were evident in 1-week HFD–BI-1 KO mice and more highly evident in 8-weeks HFD-BI-1 KO mice, compared with the 8-weeks HFD-BI-1 WT mice (Fig. 4b). ApoB hepatic accumulation is linked to an altered ER oxidative folding environment^13^. The redox status of PDI, which transfers oxidative equivalents to newly synthesized proteins, determines the ER oxidative protein folding environment^14^. In this study, the formation of heavy molecular weight complexes (HMWCs) by PDI was analyzed in non-reducing gel system. The HMWCs were prominent in the livers of 1-week HFD–BI-1 KO mice than in those of the counter-WT mice (Fig. 5a, the PDI redox status was clearly showed in separately running reduced different acrylamide gel system, middle). The complex formation was more significantly increased in 8-weeks-HFD-fed BI-1 KO mice compared with 1-week-HFD-fed BI-1 KO mice. Protein carbonylation, a type of protein oxidation, was also analyzed. PDI was found to be excessively carbonylated in the livers of 8-weeks HFD-BI-1 KO mice, with most of the oxidized PDI corresponding to the fraction residing within HMWCs (Fig. 5b). Especially, the detectable PDI amount in reduced gel system was clearly decreased (Fig. 5b, lower), suggesting that the oxidized PDI residing within the HMWC fraction from HFD-BI-1 KO mice is associated with client proteins (e.g., ApoB) that failed to fold properly. We also examined the interaction between PDI and ApoB. Whereas PDI was dissociated from ApoB in the livers of 1-week HFD-BI-1 WT mice, PDI was stably bound to ApoB in the livers of 8-weeks HFD-BI-1 KO mice, and to a lesser extent in 1-week HFD-BI-1 KO mice (Fig. 5c), suggesting that the client protein ApoB failed to fold properly and remained complexed with PDI, especially in the BI-1 KO conditions.

### BI-1 inhibits high-fat diet-induced ER stress and ROS accumulation

**Figure 6:**
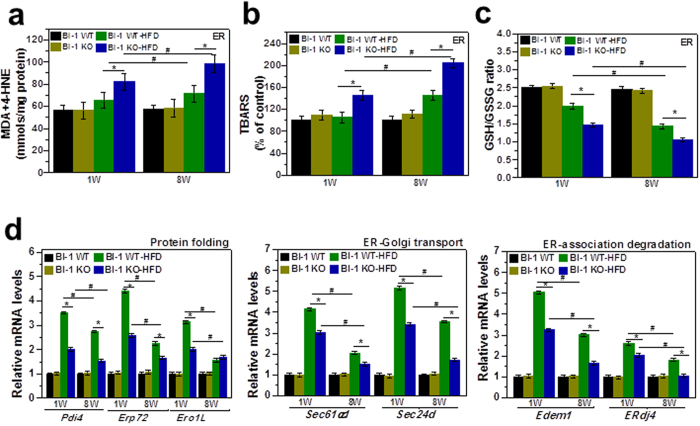
BI-1 regulates high-fat diet-induced ROS accumulation and the expression of genes involved in protein folding, ER-to-Golgi transport, ER-associated degradation, and ER stress. Malondialdehyde (MDA) and 4-hydroxynonenal (4-HNE) levels (**a**), TBARS (**b**), and GSH/GSSG ratio (**c**) were measured in the ER fractions from liver tissues of HFD-fed BI-1 WT or BI-1 KO mice. (**d**) Quantitative real-time RT-PCR analysis of liver mRNA isolated from HFD-fed BI-1 WT or BI-1 KO mice. Total liver mRNA was isolated for real-time PCR analysis. Expression values were normalized to GAPDH mRNA levels. (Mean ± SEM; n = 5, *p < 0.05, ^#^*p* < 0.01). NFD; normal-fat diet, HFD; high-fat diet, WT; wild-type, KO; knock-out, ERAD; ER-associated degradation, Pdi4, protein disulfide isomerase associated 4; *Erp72*, endoplasmic reticulum protein 72; *Ero1L*, ER oxidoreductase-1 L; *Sec61α1*, Sec61α1 subunit; *Sec24d*, SEC24-related gene family, member D; *Edem1*, ER degradation enhancing, mannosidase a-like 1; *ERdj4*, DnaJ (Hsp40) homologue, subfamily B, member 9; TBARS, Thiobarbituric acid reactive substances.

**Figure 7:**
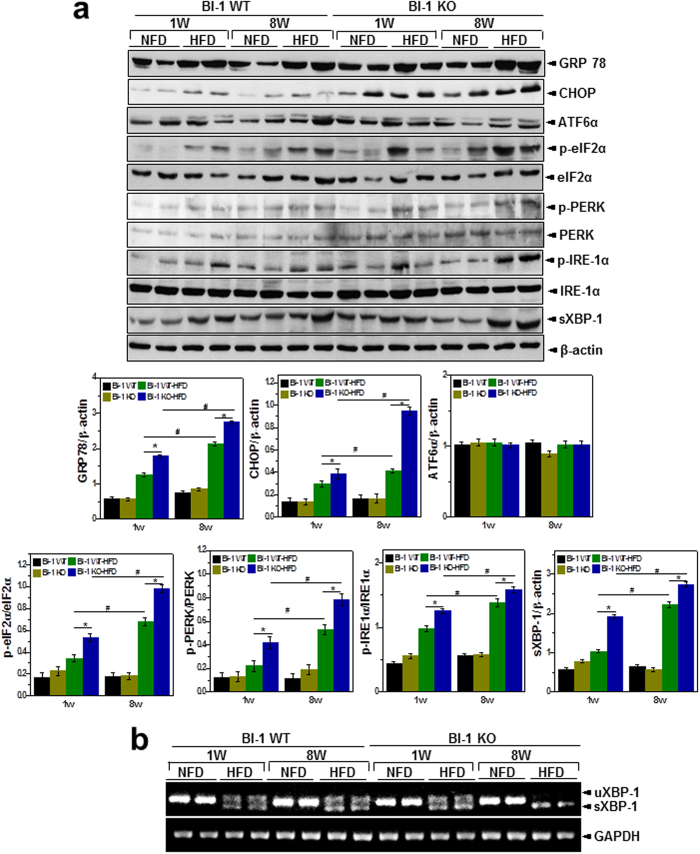
BI-1 regulates high-fat diet-induced ER stress. (**a**) From liver tissues of 1- and 8-weeks-HFD-fed BI-1 WT or BI-1 KO mice, immunoblotting was performed using antibodies against anti-GRP78, CHOP, ATF6α, p-eIF2α, eIF2α, p-PERK, PERK, p-IRE-1α, IRE1-α, sXBP-1, and β-actin. The quantification analysis about the ER stress protein expressions was performed based upon the indicated loading control (β-actin for GRP78, CHOP, ATF6α and sXBP-1, eIF2α for p-eIF2α, PERK for p-PERK and IRE1-α for p-IRE-1α, lower). (**b**) sXBP-1 was measured with RT-PCR assay as described in Experimental Procedures (Mean ± SEM; n = 5, *p < 0.05, ^#^*p* < 0.01). NFD; normal-fat diet, HFD; high-fat diet, WT; wild-type, KO; knock-out, uXBP-1; unspliced XBP-1, sXBP-1; spliced XBP-1.

It has been suggested that apolipoprotein secretion is altered under conditions of ER dysfunction, which also involve ROS accumulation^12^. Similarly, ER membrane lipid peroxidation was increased by HFD in BI-1 KO mice to a greater extent after 8-weeks of HFD feeding than after 1-week (Fig. 6a,b). To indicate ER-associated ROS, the ratio of GSH to GSSG is used as an index of the redox state^15^. As shown in Fig. 6c, the ratio of GSH to GSSG in the ER was significantly more decreased in HFD-BI-1 KO mice than in WT mice. Under the 8-week HFD-BI-1 KO condition, the ratio was more significantly decreased than under the 1-week condition. Quantitative real-time PCR analyses confirmed that BI-1 is required for genes encoding mediators of protein folding functions such as cis-trans proline isomerization and disulfide bond formation (*Pdi4, Erp72*, and *Ero1L*), ER-to-Golgi protein transport (*Sec61α1* and *Sec24d*) and ER-associated degradation (ERAD) (*Edem1* and *ERdj4*). Under HFD stress, ER folding function genes were highly increased, especially in the 1-week HFD BI-1 WT group, indicating an adaptive and physiological response against transient HFD stress (Fig. 6d). Under the longer stress period (8 weeks), gene expression was increased to a lesser extent. Increases in ER folding function gene expression were also significantly lower in the BI-1 KO condition compared with the WT condition. To examine the ER stress response induced by a HFD, we analyzed the expression of UPR proteins in 1- and 8-weeks HFD-BI-1 KO mice. As expected, GRP78, CHOP, p-eIF2α, p-PERK, p-IRE-1α, and spliced XBP-1 were more significantly increased in liver tissue from 8-weeks HFD–BI-1 KO mice compared with tissue from 1-week HFD-BI-1 KO mice (Fig. 7a,b).

### Hepatic BI-1 expression relieves ApoB accumulation and its related formation of disulfide bonds in high-fat diet-fed BI-1 knock-out mice

**Figure 8:**
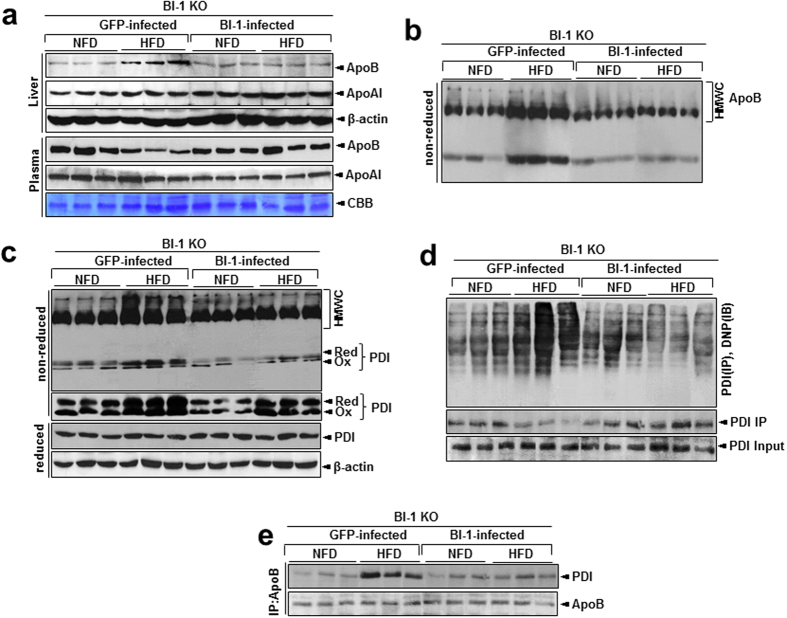
Hepatic BI-1 expression regulates dyslipidemia and the formation of disulfide bonds during oxidative protein folding in high-fat diet-fed mice. (**a**) In 1-week-HFD-fed BI-1 WT mice that were infected with GFP control virus and BI-1-expressing virus, liver and plasma samples were subjected to immunoblot analysis with anti-ApoB or anti-ApoA-I antibody. CBB staining (albumin) was performed as a loading control. (**b**) Liver lysates from the mice were analyzed for the presence of ApoB in high molecular weight complexes (HMWCs) on non-reducing gels. (**c**) Whole livers from mice fed a normal diet or HFD and infected with GFP control virus and BI-1-expressing virus were analyzed for the presence of PDI in HMWCs with non-reducing (top and 2^nd^ panels) and reducing gels (3^rd^ and 4^th^ panels). (**d**) Liver lysates were derivatized with DNPH, and PDI was immunoprecipitated from derivatized lysates. Immunoprecipitated PDI was run on a non-reducing gel and analyzed by immunoblotting using anti-DNP antibody. Note that the main 57-kDa band is weaker in lysates from HFD-fed Ad GFP mice than HFD-fed Ad BI-1 mice. (**e**) Liver lysates were immunoprecipitated with anti-ApoB or PDI antibody and immunoblotted with anti-PDI or ApoB antibody. NFD; normal-fat diet, HFD; high-fat diet, WT; wild-type, KO; knock-out, PDI; protein disulfide isomerase, HMWCs; high molecular weight complexes, CBB; coomassie brilliant blue.

The role of BI-1 was further examined through adenoviral BI-1 hepatic infection of BI-1 KO mice. The expression of ApoA-I and ApoB was analyzed after feeding HFD to BI-1 KO mice, infected separately with Ad GFP and Ad BI-1 virus, 4 days later. In liver of Ad GFP, the expression of ApoB was increased and ApoA-I was not affected under HFD feeding (Fig. 8a). Ad BI-1 infection modulated the change in hepatic ApoB expression in mice fed HFD. Specifically, the level of secreted ApoB in the plasma decreased upon HFD feeding in Ad GFP-infected mice, and was recovered in Ad BI-1 mice. As shown in Fig. 8b, ApoB accumulation in HMWCs was observed in the HFD-Ad GFP mice, but ApoB aggregate forms were not found in Ad BI-1 mice. We tested whether HMWC formation is regulated by BI-1 by analyzing complexes formed by PDI using non-reducing gels. HMWCs were less prominent in HFD-Ad BI-1 mice than in HFD-Ad GFP mice (Fig. 8c). Whereas the redox status of PDI was highly increased in HFD-Ad GFP mice, it was significantly regulated in the presence of BI-1. Expression of BI-1 clearly reduced the large accumulation of carbonylation in the liver tissue after HFD feeding (Fig. 8d). We also examined the interaction between PDI and ApoB. PDI was bound to ApoB in liver tissue from HFD-Ad GFP mice but the PDI was easily dissociated from ApoB in the liver tissue of HFD-Ad BI-1 mice (Fig. 8e).

### Hepatic BI-1 expression inhibits high-fat diet-induced ER stress and ROS accumulation

**Figure 9:**
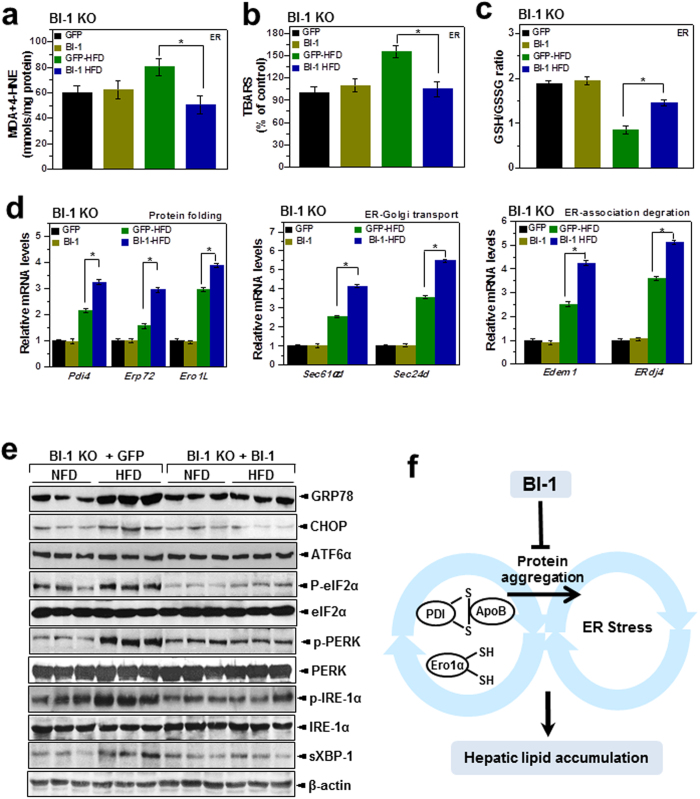
Hepatic BI-1 expression inhibits high-fat diet-induced ROS accumulation, controlling the expression of genes involved in protein folding, ER-to-Golgi transport, ER-associated degradation, and ER stress. In the liver tissues of HFD-fed BI-1 WT mice infected with GFP control virus and BI-1-expressing virus, ER was fractionized. With the ER fractions, hepatic MDA and 4-HNE levels (**a**), TBARS (**b**), and GSH/GSSG ratio (**c**) were measured. (**d**) For the indicated gene analysis, quantitative real-time RT-PCR was performed. Expression values were normalized to GAPDH mRNA levels. (**e**) Immunoblotting was performed using antibodies against GRP78, CHOP, ATF6α, p-eIF2α, eIF2α, p-PERK, PERK, p-IRE-1α, IRE1-α, sXBP-1, and β-actin. (**f**) Schematic diagram of ROS involvement and protein aggregation in HFD-induced lipid accumulation. (Mean ± SEM; n = 5, *p < 0.05). MDA, malondialdehyde; 4-HNE, 4-hydroxynonenal; TBARS, Thiobarbituric acid reactive substances.

The expression of hepatic BI-1 reduced ER membrane lipid peroxidation after HFD-feeding (Fig. 9a,b). As shown in Fig. 9c, expression of BI-1 recovered the reduced ratio of GSH to GSSG in the ER in animals fed HFD. Quantitative real-time PCR analyses confirmed that the ER function genes encoding mediators of protein folding such as cis-trans proline isomerization and disulfide bond formation (*Pdi4, Erp72*, and *Ero1L*), ER-to-Golgi protein transport (*Sec61α1* and *Sec24d*), and ER-associated degradation (ERAD) (*Edem1* and *Erdj4*) were significantly increased in the Ad BI-1-infected groups (Fig. 9d). Expression of BI-1 also reduced the high activation level of ER stress proteins including GRP78, CHOP, p-eIF2α, p-PERK, p-IRE-1α, and spliced XBP-1 in liver tissues under HFD-feeding conditions (Fig. 9e). As an important protein disulfide bond formation chaperone, PDI status clearly demonstrates the role of BI-1 as an enhancer of the protein folding capacity linked to the maintenance of lipid homeostasis (Fig. 9f).

## Discussion

In this study, we examined the effect of BI-1 on the modification of oxidative intra-ER protein folding in a high-fat diet model. BI-1 regulates ER stress and the resultant hepatic lipid accumulation *in vivo* through maintenance of the levels of hepatic ApoB and secretion of TG and cholesterol. Furthermore, intra-ER ROS production was correlated with ER stress, and was also regulated in the presence of BI-1. After HFD exposure, PDI was found to be predominantly complexed with client proteins (e.g., ApoB), reflecting the inability of PDI to assist in disulfide bond formation; this complex formation was rather enhanced in the absence of BI-1. Therefore, BI-1 regulates HFD-associated ROS generation and affects the ER folding environment, including the PDI redox status.

### BI-1 regulates lipid secretion in a high-fat diet exposed NAFLD model

In the 1-week model, a relatively short-term stress induced by high-fat diet feeding, the lipid synthesis pathway was not affected but ApoB, a key apolipoprotein for transfer of TG and cholesterol, was highly affected. Intrahepatic lipid accumulation without plasma lipid accumulation was observed in the 1-week HFD-fed mice, especially in the ER stress-susceptible BI-1 knock-out mouse model (Figs 1a,b and 2a,b). Compared with short-term stress, the 8-week model showed a highly activated fatty acid synthesis pathway (Fig. 3a). It was recently suggested that chronic feeding with HFD stress induces SREBP-1c transcriptional activation, amplifying the downstream lipid synthesis profile genes^16^,^17^. However, it has been also reported that transient/short-term HFD exposure significantly induces hepatic lipid accumulation without an effect on lipid synthesis^18^. The transient feeding diet did not alter the expression of several key genes involved in cholesterol biosynthesis, such as sterol regulatory element binding protein 2 (SREBP-2). Instead, short-term HFD stress seems to be related more to alterations in protein secretion, rather than enhancement of lipid synthesis. In conditions of chronic HFD stress, an enhanced requirement for lipid synthesis is presumed to increase the protein-folding burden on the ER, thus the ER stress response is greater for chronic HFD than for short-term HFD conditions. Throughout this study, BI-1 was shown to control ER stress and its related dyslipidemia associated with both conditions.

### PDI-associated alterations in the ApoB folding/secretion process in the NAFLD model are regulated by BI-1

Accumulation of Apo B was enhanced in the HFD-BI-1 KO model, compared with the WT model. Moreover, ApoB was more accumulated in the condition of chronic HFD-feeding over 8 weeks (Fig. 4a). Recent studies implicating hepatic ER stress as a central abnormality linking obesity, hepatic insulin resistance, and hepatic steatosis have added a new level of complexity to this issue^19^,^20^,^21^. For example, ER stress has been also linked to increased hepatic lipogenesis^22^,^23^. Additionally, secretory complex proteins such as ApoB might be a prime target for ER stress-induced protein misfolding; functionally altered protein. Thus, ER stress–mediated reductions in ApoB secretion could be a link between ER stress and hepatic lipid accumulation. Consistent with these findings, it has been also reported that substantial accumulation of hepatic lipids is directly related with ER stress^24^,^25^.

Data presented in this study suggest that the ER stress-mediated reduction in ApoB secretion is also related to dysfunctional PDI chaperone activity, a fundamental assisting system for protein folding^26^. The unspecified folding proteins associated with the chaperone protein PDI was clearly accumulated as an aggregated form with the client proteins, especially in the BI-1 KO mice (Figs 5a and 8c), indicating that the BI-1 KO condition leads to ineffective function of chaperones and isomerases such as PDI. We also showed that carbonylated proteins were highly associated with PDI, especially in the BI-1 KO mice (Figs 5b and 8d), suggesting that the majority of oxidized PDI resides within the heavy molecular weight complex fraction in association with client proteins. If PDI works effectively, it would not reside within the heavy molecular weight complex fraction in association with client proteins, but would release the folded client proteins to the appropriate subcellular organelles. This study shows reduced/altered function of protein folding capacity, especially in the HFD-fed BI-1 KO condition.

For effective folding of client proteins including ApoB, chaperone proteins including PDI must be easily dissociated from the client proteins. However, in the BI-1 KO condition, the association between PDI and ApoB was tighter and more stable, compared with the BI-1 WT conditions (Figs 5c and 8e). These findings reflect the inability of PDI to assist in disulfide bond formation in the HFD-treated BI-1 KO model. During exposure to HFD, abnormal oxidation of PDI by HFD-induced ROS may be a primary cause of the ER stress response in BI-1 KO mice. Previously, the ER stress-associated ROS has been suggested to be CPR-CYP-coupling system hyperactivation or alteration^7^,^27^. In this study, CYP2E1 was observed to be highly induced under both the 1- and 8-week HFD conditions (Supplementary Fig. 2). Although BI-1 interacts with CPR disturbing the CPR/CYP redox, the direct interaction of BI-1 with PDI family proteins has not been observed (data not shown). The CYP2E1 hyperactivation and its coupling ROS production may contribute to the abnormal oxidation of PDI, especially in the HFD-treated BI-1 KO mice. Throughout this study, HFD stress is linked to the PDI alterations/ER stress-associated ROS accumulation in the ApoB folding/secretion process, which is regulated by BI-1.

### BI-1 regulates lipid metabolism through ER stress regulation

For adaptation against HFD-induced stress, the folding capacity needs to be increased through ER chaperones and enzymes that are required for protein oxidative folding, ER-to-Golgi trafficking, and *ERAD. Pdi4, Erp72*, and *Ero1L* for protein folding, *Sec61α1*, and *Sec24d* for ER-to-Golgi trafficking, and *Edem1* and *Erdj4* for ERAD were highly induced in the HFD-fed condition (Figs 6d and 9d). This expression pattern can be explained as a response to protein folding stress to manage the abnormal/unfolding protein-folding condition. Compared with BI-1 WT mice, the genes involved in ER folding were less strongly induced in the BI-1 KO mice, suggesting that ER folding capacitance is downregulated in the absence of BI-1. The ER-functional gene analysis seems to be different from that of the classic UPR^28^, which is also an adaptation process against ER stress. However, the above-mentioned genes-*Pdi4, Erp72, Ero1L, Sec61α1, Sec24d, Edem1*, and *Erdj4* are more strongly related to the ER environment through ER redox or ER calcium buffering capacity than to cytosolic signaling transduction (i.e., IRE-1α, PERK, and ATF6 are ER stress markers for inducing cytosolic signaling)^28^,^29^,^30^,^31^,^32^,^33^. Considering that the ER serves as a protein-folding factory where elaborate quality and quantity control systems monitor the efficient and accurate production of secretory and membrane proteins and constantly maintain proper physiological homeostasis within the ER, including redox state and calcium balance, ER functional genes including *Pdi4, Erp72, Ero1L, Sec61α1, Sec24d, Edem1*, and *Erdj4* most likely represent ER folding capacity. The PDI chaperone capacity (Figs 5b,c and 8d,e) is also consistent with this notion. It might be suggested that BI-1 functions as a kind of reservoir that effectively covers the ER folding requirement. Consistent with this, the enhanced folding capacity of BI-1 for a specific protein, V-ATPase, has already been reported in another pathological condition, lung fibrosis^34^. Considering that BI-1 enhances ER folding capacity, the KO condition may well lead to upregulation of UPR proteins including GRP78, CHOP, p-eIF2α, p-PERK, p-IRE-1α, and sXBP-1 under HFD stress conditions (Fig. 7a,b).

The UPR is now recognized to have important physiological roles in metabolism, inflammation, and cell differentiation and survival^35^,^36^,^37^. In the liver, ER stress and UPR signaling can be triggered by metabolic factors, such as lipids, glucose, cytokines, homocysteine, and free fatty acids^38^. Consistent with this, regulation of ER stress is suggested as a major mechanism underlying the BI-1-induced effect against lipid dysmetabolism demonstrated in this study.

In summary, we found that BI-1 regulates HFD-induced ER stress and consequent ROS accumulation. Our findings provide molecular evidence supporting the use of BI-1 as a possible therapeutic agent for the management of hepatic dyslipidemia.

## Methods

### Reagents

*N*-ethylmaleimide (NEM) was purchased from Sigma-Aldrich (St. Louis, MO, USA). The following primary antibodies were used: p-eIF2α, IRE-1α (Cell Signaling Technologies, Danvers, MA, USA); GRP78, CHOP, ATF6α, PERK, p-PERK, sXBP-1, and β-actin (Santa Cruz Biotechnologies, Inc., Santa Cruz, CA, USA); p-IRE-1α (Abcam, Cambridge, MA, USA) and monoclonal PDI (clone 1D3, Enzo Life Sciences, Farmingdale, NY, USA). HRP-conjugated secondary antibodies (anti-mouse, anti-rabbit, and anti-rat) were obtained from Santa Cruz Biotechnologies. Kits for the analysis of triglyceride (TG) and cholesterol were purchased from Asan Medical (Seoul, S. Korea). The ApoB 100 kit was purchased from Kamiya Biomedical Co. (Seattle, WA, USA). All other chemicals were purchased from Sigma.

### Animal experiments

BI-1 WT and BI-1 KO C57BL/6 male mice (n = 120 each, 8 weeks old) were housed in groups of 5 animals at at 21 ± 1 °C and 55 ± 5% humidity under a 12 h/12 h light/dark cycle. Mice were maintained in specific pathogen-free housing and were cared for in accordance with the regulations of the Care and Use of Laboratory Animals guide of Chonbuk National University and were approved by the Chonbuk National University laboratory animal center of the Institutional Animal Care and Use Committee (IACUC, CBNU 2015-0002).

### Adenovirus-mediated gene transfer *in vivo*

The adenoviral vector expressing BI-1 (Ad BI-1) was generated using the Gateway system (Invitrogen, Carlsbad, CA, USA). Sequence-specific recombination between the BI-1 expression plasmid cassette and the adenovirus backbone plasmid pAd/PL-DEST^TM^ (Invitrogen) was carried out using the Gateway^®^ LR Clonase^TM^ enzyme mix (Invitrogen). Linearized recombinant adenoviral genomes were transfected into 293 cells allowing for the growth of recombinant adenoviruses. Adenoviral stocks were purified twice by CsCl gradient centrifugation, dialyzed extensively against virus storage buffer (137 mM NaCl, 5 mM KCl, 10 mM Tris, pH 7.4, 1 mM MgCl_2_), and stored in small aliquots at −80 °C until further use. Titers of purified adenoviral vectors were determined as plaque-forming units (PFUs) by immunofluorescence staining using fluorescein isothiocyanate-conjugated anti-Ad5-hexon antibody K6100 (DakoCytomation, Glostrup, Denmark). For adenovirus-mediated gene transfer *in vivo*, BI-I KO mice on a HFD and normal-diet mice were anesthetized with ketamine and were infected via a single injection of purified 1.0 × 10^9^ PFU (diluted in 0.1 ml saline) in a volume of 50 μL of Ad-GFP or Ad-BI-1 adenoviruses via the tail-vein, as previously described^39^. Mice were sacrificed on day 4 after inoculation; blood samples were collected and livers were snap frozen in liquid nitrogen and stored at −80 °C for subsequent analysis.

### Animal Sacrifice

Mice were anaesthetized with diethyl ether (Sigma) and sacrificed by cervical dislocation. Tissues and blood samples were collected from all sacrificed animals. Whole blood was immediately placed on ice in a 1.5 mL centrifuge tube for 15 to 30 min and spun at 8,000 rpm for 10 min. Sera were then transferred to fresh 1.5 mL centrifuge tubes and stored at −80 °C. All harvested tissues were immediately placed in liquid nitrogen and stored at −80 °C.

### Immunoblotting

Liver tissue homogenates were prepared by homogenizing the tissue in lysis buffer (20 mM Tris, pH 7.4; 150 mM NaCl; and 0.5% Triton X-100) or RIPA buffer (150 mM NaCl; 50 mM Tris, pH 8.0; 2 mM EDTA; 1% Nonidet P-40; and 0.1% SDS) supplemented with a protease inhibitory cocktail tablet (Roche, Indianapolis, IN, USA) and phosphatase inhibitory cocktails 2 and 3 (Sigma-Aldrich). Lysates were cleared by centrifugation and analyzed by gel electrophoresis. Protein concentration was determined via Bradford protein assay (Bio-Rad, Hercules, CA, USA) using BSA as a standard, and verified by Coomassie blue gel staining. Lysates (45 μg) were resolved by SDS-PAGE (Bio-Rad) and then transferred to nitrocellulose membranes. Membranes were blocked for 1 h with 5% skim milk in Tris-buffered saline (0.137 M NaCl, 0.025 M Tris, pH 7.4) containing 0.1% Tween-20 (T-TBS). Antibodies were diluted according to the manufacturers’ recommended protocols. Protein signals were visualized using an enhanced chemiluminescence (ECL) reagent (SuperDetect^TM^ ECL Western Blotting Detection Reagent, DaeMyung Science Co., Ltd, Seoul, Korea). Finally, membranes were exposed to imaging film (Kodak BioFlexEcono Scientific Supplies, Citrus Heights, CA, USA), which was developed using a Kodak X-OMAT 1000A Processor.

### Immunoprecipitation

For immunoprecipitation, mouse liver lysates were prepared in 50 mM Tris-HCl, pH 8.0, containing 150 mM NaCl, 0.015% phenylmethylsulphonyl fluoride, 1 mM dithiothreitol, 1 mM EDTA, 1% sodium deoxycholate, 1% Triton X-100, and 1% SDS. Liver lysates were incubated at 70 °C for 15 min to ensure complete cell lysis, and were then diluted with lysis buffer to achieve a SDS concentration of 0.1% (w/v). Lysates were incubated with antibodies (1:100 dilution) for 1 h and then with 20 μL of protein A/G-Sepharose (10% solution) for an additional 2 h. Immunocomplexes were collected by centrifugation, washed three times with 10 mM Tris, pH 7.5 containing 0.1 M NaCl and 1% Triton X-100, eluted in 50 μL of sample buffer, separated on SDS–polyacrylamide gel, and exposed to a phosphoimaging screen to visualize the results.

### Oil red O staining

To measure neutral lipid droplet accumulation, mouse liver cryosections were fixed for 10 min in 60% isopropanol, stained with 0.3% oil red O in 60% isopropanol for 30 min, and washed with 60% isopropanol. Sections were counterstained with Gill’s hematoxylin, washed with acetic acid solution (4%), and then mounted in aqueous solution. Slides were photographed with an Eclipse TE2000 inverted microscope system (Nikon Instruments, Melville, NY, USA) at x200 magnification.

### Measurement of total lipid, triglyceride, and cholesterol levels

To measure the lipid levels, mouse liver homogenates were extracted according to a modified Bligh and Dyer procedure^40^. Samples were homogenized with chloroform-methanol-water (8:4:3), shaken at 37 °C for 1 h, and centrifuged at 1,100 × *g* for 10 min. The bottom layer was collected and suspended for hepatic lipid analysis. Triacylglycerol, total cholesterol, and total lipid contents were measured using kits from Randox Laboratories (Antrim, UK) in accordance with the manufacturer’s instructions.

### Biochemical evaluation

Blood samples were collected in tubes at specific time points. After centrifugation at 2,000 × *g* for 10 min, triglyceride and cholesterol were measured using kits according to the manufacturer’s instruction.

### PDI redox state and high-molecular-weight protein complex formation in the liver samples

Procedures were performed as described previously^41^. Briefly, liver tissue was washed twice with ice cold PBS supplemented with 20 mM NEM to protect the reduced disulfide bonds from further oxidation during lysis, lysed in lysis buffer (20 mM Tris, pH 7.4; 150 mM NaCl; and 0.5% Triton X-100) for 30 min on ice, and cleared by centrifugation. The protein concentration was determined by the Bio-Rad Bradford protein assay using BSA as a standard; 50 μg of the total protein was separated in non-reducing polyacrylamide gels (no SDS, not boiled). 12% polyacrylamide gels were run at 4 °C at 50 V to distinguish between redox forms of PDI. 8% polyacrylamide gels were used to detect complexes between PDI and its client proteins.

### Detection of carbonylated PDI

Proteins (100 μg) from liver tissue homogenates were derivatized for 5 min using the OxyBlot kit (Millipore, Billerica, MA, USA). The reaction was stopped by the addition of a neutralization solution as per the manufacturer’s instructions. To remove 2, 4-dinitrophenylhydrazine (DNPH), proteins were pelleted by ultracentrifugation (Beckman TL-100 ultracentrifuge, TLA100.2 rotor; Beckman Coulter, Fullerton, CA, USA) at 100,000 × *g* for 1 h at 4 °C. Protein pellets were washed with IP lysis buffer and resuspended in 500 μL of IP-lysis buffer. PDI was immunoprecipitated as described above, resolved in non-reducing gels, Western blotted, and probed with anti-DNP rabbit antibodies as per the OxyBlot kit instructions.

### ER fractionation

The microsomal fraction was obtained as previously described^42^. Briefly, liver tissues were resuspended in buffer A (250 mM sucrose, 20 mM HEPES, pH 7.5, 10 mM KCl, 1.5 mM MgCl2, 1 mM EDTA, 1 mM EGTA, and protease inhibitor complex (Roche Diagnostics, Mannheim, Germany) on ice for 30 min. The lysates were then homogenized and centrifuged at 750 × g for 10 min at 4 °C. The supernatant from the homogenized lysates was then centrifuged at 100,000 × g for 1 h at 4 °C. The resulting supernatant was discarded, and the pellet was saved at −75 °C.

### Measurement of lipid peroxidation

To measure ER membrane lipid peroxidation, the concentrations of the lipid peroxidation products, malondialdehyde (MDA) and 4-hydroxynonenal (4-HNE), were measured using the BIOXYTECH LPO-586 commercial kit (Oxis International Inc., Portland, OR, USA) according to the manufacturer’s protocol. The reactive aldehydic products of lipid peroxidation, MDA and 4-HNE, were measured in duplicate and expressed as nmol/mg of protein^43^. Separately, lipid hydroperoxide was measured using an LPO assay (No. 705003) (Cayman Chemical, Ann Arbor, MI, USA) according to the manufacturer’s protocol. Lipid hydroperoxide was measured in triplicate.

### Gene expression using quantitative PCR

Total RNA was extracted and reverse transcribed into cDNA using 1 μg RNA. The primers used to quantify lipogenesis have previously been described^44^,^45^. The sequences of the primers used for RT-PCR were as follows: mouse FAS: 5′-CCCAGCCCATAAGAGTTACA-3′ (forward); 5′-ATCGGGAAGTCAGCACAA-3′ (reverse); mouse SCD1: 5′-GCTCTACACCTGCCTCTTC-3′ (forward); 5′-CCGTGCCTTGTAAGTTCTG-3′ (reverse); mouse ACC1: 5′-GCAACCACATCTTCCTCAACT-3′ (forward); 5′-CACAGACGGCTGCCATAG-3′ (reverse); mouse HMGCS: 5′-TTTGACCGAGGGCTCCGTGG-3′ (forward); 5′-TCCAGGGCGCTGAGGTAGCA-3′ (reverse); mouse HMGCR: 5′-TGTGGGAACGGTGACACTTA-3′ (forward); 5′-CTTCAAATTTTGGGCACTCA-3′ (reverse); mouse Pdia4: 5′-AAGTCTGAACCCATCCCAGAGT-3′ (forward); 5′-TATGTCATCAAAATTCTCTGCTACCA-3′ (reverse); mouse Erp72: *5*′**- CAGCTCTGCACCCAATCATG-3′ (forward); 5′CCTCCTCAGTGGCATTTTCTGT-3′ (reverse); mouse Ero1L: 5′-TCAGTGGACCAAGCATGATGA-3′ (forward); 5′-TCCACATACTCAGCATCGGG-3′ (reverse); mouse Sec61α1: 5′-CTATTTCCAGGGCTTCCGAGT-3′ (forward); 5′-AGGTGTTGTACTGGCCTCG-3′ (reverse); mouse Sec24d: 5′-CTCATATAGAAAGCGCCAAAGAAAT-3′ (forward); 5′-TCATGTACACAGGCAGCACCTT-3′ (reverse); mouse Edem1: 5′-AAGCCCTCTGGAACTTGCG-3′ (forward); 5′-AACCCAATGGCCTGTCTGG-3′ (reverse); mouse ERdj4: 5′-CCCCAGTGTCAAACTGTACCAG-3′ (forward); 5′-AGCGTTTCCAATTTTCCATAAATT-3′ (reverse); mouse GAPDH: 5′-GGATTTGGCCGTATTGGG-3′ (forward); 5′-GTTGAGGTCAATGAAGGGG-3′ (reverse). Quantitative PCR was run in triplicate using iQSYBR Green Supermix (Bio-Rad, Hercules, CA, USA) and an iCycler (Bio-Rad) with 40 cycles of amplification (95 °C for 10 s) and annealing (58 °C for 30 s). The amplified products were confirmed via gel electrophoresis and melt curve analysis. Results were calculated by a 2^-ΔΔCt^ method, and presented using chow groups as 100%.

### Statistical analysis

All experimental data are shown as the mean ± SEM. Statistical significance was determined by one-way ANOVA and Tukey’s post-tests. Statistics were calculated with Origin (Origin-Lab Corporation, Northampton, MA, USA), and significance was indicated when *p* < 0.05.

## Additional Information

**How to cite this article**: Lee, H. Y. *et al*. Bax Inhibitor-1 regulates hepatic lipid accumulation via ApoB secretion. *Sci. Rep.* **6**, 27799; doi: 10.1038/srep27799 (2016).

## Supplementary Material

Supplementary Information
