# Author Correction: Bax Inhibitor-1 regulates hepatic lipid accumulation via ApoB secretion

**DOI:** 10.1038/s41598-023-49504-4

**Published:** 2023-12-21

**Authors:** Hwa Young Lee, Geum-Hwa Lee, Kashi Raj Bhattarai, Byung-Hyun Park, Seung-Hoi Koo, Hyung-Ryong Kim, Han Jung Chae

**Affiliations:** 1 Department of Pharmacology, School of Medicine, Chonbuk National University, Jeonju, 560-182 Korea; 2 Department of Biochemistry, School of Medicine, Chonbuk National University, Jeonju, 560-182 Korea; 3 Division of Life Sciences, Korea University, 145 Anam-Ro, Seongbuk-Gu, Seoul, 136-713 Korea; 4 Department of Dental Pharmacology, School of Dentistry, Wonkwang University, Iksan, 570-749 Korea

Correction to: *Scientific Reports* 10.1038/srep27799, published online 14 June 2016

This Article contains an error in Figure. 2, where an inadvertent duplication of images occurs. The correct Figure. 2 and accompanying legend appear below.

**Figure 2:**
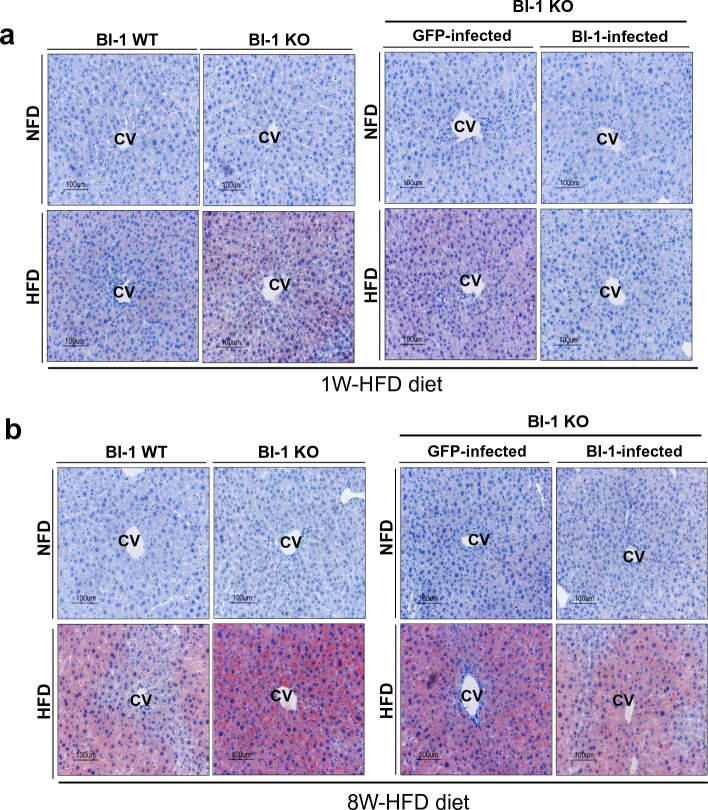
(**a**) BI-1 WT and BI-1 KO mice (8 weeks old, n = 5) were fed with HFD for 1-week and separately, 1-week-HFD-fed BI-1 KO mice were infected with GFP control virus and BI-1-expressing virus (right). (**b**) BI-1 WT and BI-1 KO mice (8 weeks old, n = 5) were fed with HFD for 8-weeks and separately, 8-weeks-HFD-fed BI-1 KO mice were infected with GFP control virus and BI-1-expressing virus (right). Oil Red O staining was performed. Images of liver samples were obtained at 200 × original magnification. CV; central vein, NFD; normal-fat diet, HFD; high-fat diet, WT; wild-type, KO; knock-out, GFP; green fluorescent protein.

