# Supplementary material for: Bax Inhibitor-1 regulates hepatic lipid accumulation via ApoB secretion
Source: Sci Rep. 2016 Jun 14;6:27799. doi: 10.1038/srep27799 (PMC4906294; doi:10.1038/srep27799)

Bax Inhibitor-1 regulates hepatic lipid accumulation via ApoB secretion

Hwa Young Lee[1], Geum-Hwa Lee[1], Kashi Raj Bhattarai[1], Byung-Hyun Park[2], Seung-Hoi Koo[3], Hyung-Ryong Kim[4¶], Han Jung Chae[1¶]

[1]Department of Pharmacology, School of Medicine, Chonbuk National University, Jeonju 560-182, Korea, [2]Department of Biochemistry, School of Medicine, Chonbuk National University, Jeonju 560-182, Korea, [3]Division of Life Sciences, Korea University, 145 Anam-Ro, Seongbuk-Gu, Seoul, 136-713, Korea, [4]Department of Dental Pharmacology, School of Dentistry, Wonkwang University, Iksan, 570-749, Korea.

[¶]To whom correspondence should be addressed: Han-Jung Chae, Department of Pharmacology, School of Medicine, Chonbuk National University, Jeonju, Korea; Tel: 82-63-270-3092; Fax: 82-63-275-2855; Email:hjchae@jbnu.ac.kr, Hyung-Ryoung Kim, DDS, PhD, Department of Dental Pharmacology and Working Dental Research Institute, School of Dentistry, Wonkwang University, Iksan, Chonbuk. Tel: 82-63-854-0285; E-mail: hrkimpd@wonkwang.ac.kr.

Running title: BI-1 regulates acute hepatic lipid accumulation

Supplemental Fig. 1. AST and ALT in plasma were measured at the indicated times.

BI-1 WT or BI-1 KO male C57/BL mice (8 weeks old, n=5) were fed a normal feeding diet or high-fat diet for 1 or 8 weeks. (a) AST and ALT test were measured in BI-1 WT or BI-1 KO mice. (b) AST and ALT test were measured from 1-week high fat diet-fed mice that were infected with either GFP control virus or BI-1 expressing virus.

Supplementary Fig. 2. BI-1 inhibits high-fat diet-induced CYP2E1 and CPR activity. CYP2E1 2E1 and CPR activity were analyzed in liver lysates.

Supplementary Fig 1

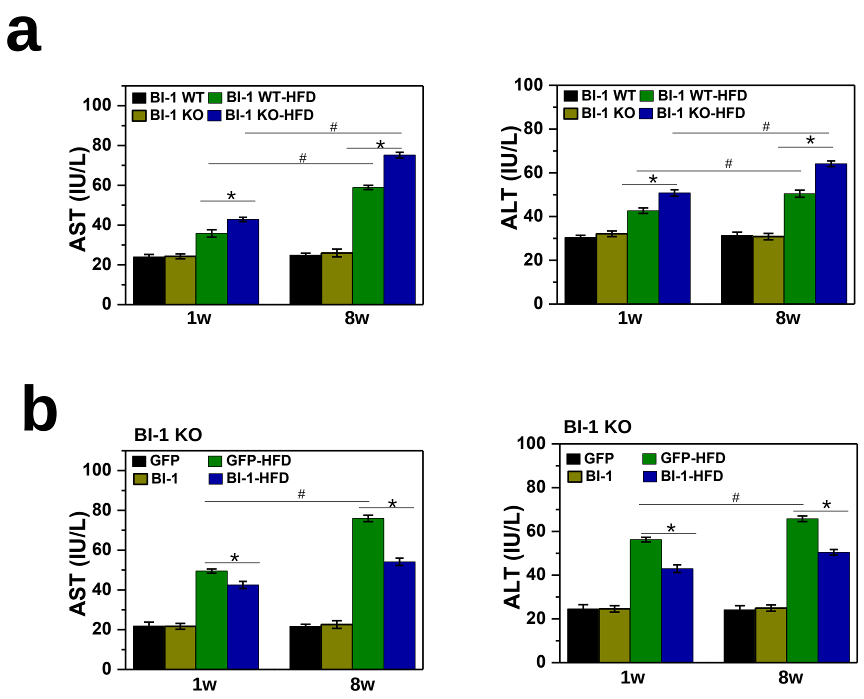

Supplementary Fig 2

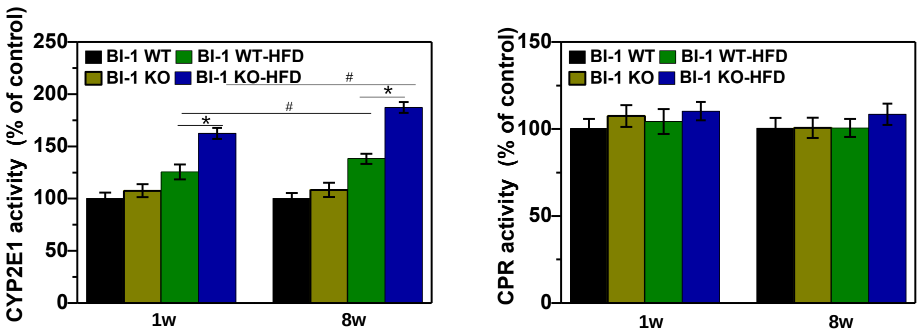

Supplement: Supplementary Information [file srep27799-s1.pdf]
